# Immunophenotypes of Newborns From SARS-CoV-2-infected Mothers

**DOI:** 10.1097/INF.0000000000004289

**Published:** 2023-12-22

**Authors:** Marta Stracuzzi, Laura Paradiso, Simona Panelli, Antonella Amendola, Elisabetta Tanzi, Clara Fappani, Gianvincenzo Zuccotti, Vania Giacomet

**Affiliations:** * From the Department of Pediatrics, Paediatric Infectious Disease Unit, Luigi Sacco Hospital; † Department of Biomedical and Clinical Sciences “L. Sacco,” Pediatric Clinical Research Center “Romeo ed Enrica Invernizzi”; ‡ Department of Health Sciences; § EpiSoMI CRC-Coordinated Research Centre, Università degli Studi di Milano, Milan, Italy; ¶ Department of Clinical Sciences and Community Health; ∥ Department of Pediatrics, V. Buzzi Children’s Hospital, Università degli Studi di Milano, Milan, Italy.

**Keywords:** pediatrics, SARS-CoV-2, newborns, immune response, lymphocyte

## Abstract

**Background:**

Little is known about the neonatal immunologic response to a maternal SARS-CoV-2 infection present during childbirth. Here we analyze a cohort of 75 neonates from SARS-CoV-2-infected mothers.

**Methods:**

The SARS-CoV-2 infection status was laboratory assessed by real-time reverse transcription polymerase chain reaction on nasopharyngeal swabs (NPS) in both mothers during childbirth and neonates within 24 hours of life. Immunophenotypes of peripheral blood mononucleated cells and SARS-CoV-2 antispike IgA, IgM and IgG of the newborns were recorded. Ten (13.3%) of 75 neonates had positive NPS for SARS-CoV-2; 17 of 75 (23%) were SARS-CoV-2-IgG seropositive, of which one with positive NPS. All the newborns resulted seronegative for SARS-CoV-2 IgA and IgM and were asymptomatic. Our cohort of newborns was divided into groups according to IgG seropositivity (IgG+/−) and NPS results (NPS+/−).

**Results:**

The count and proportion of lymphocyte subsets (evaluated measuring CD3, CD4, CD8 and CD19 markers) and of natural killer cells (evaluated by measuring the CD3−/CD16+/CD56+ subset) were all in the normal range, with no statistical differences among groups. We found a significant expansion of the T cell (CD3+) subset in the IgG+ group interpreted as the result of immune effects triggered by trained immunity in these newborns, but a decrease in CD4+ T cells for NPS+ neonates. It is therefore difficult to conclude that the decrease in CD4 can certainly be caused by an infection.

**Conclusions:**

A maternal SARS-CoV-2 infection resulted in an expansive effect of CD3+ T cells in IgG+ newborns; nonetheless, it seems not to affect structural and functional development of the newborn immune system.

Infections acquired during pregnancy can affect the differentiation and functioning of the newborn immune system in different ways and lead to both positive and negative outcomes.^1^ For example, exposure to maternal chorioamnionitis has been shown to reduce the risk of late-onset sepsis in preterm infants: a positive effect of perinatal inflammation in enhancing the functional maturation of the preterm immune system has been proposed to explain this observation.^2^ Conversely, clear relationships appear to link chorioamnionitis with a variety of other untoward neonatal morbidities: the same enhanced innate immune responses that follow this exposure are thought to negatively contribute to these sequelae of preterm birth.^3^

In general, it is increasingly recognized that in utero exposure to a variety of pathogenic agents can shape neonatal immunity, by inducing complex changes that prime its functional maturation.^4^ This may happen via the induction of a state of trained immunity, which, through an enhanced innate immune cells maturation, leads to modifications in the cytokine environment and in subsets of adaptive immunity (eg, in terms of T helper, Th, 1 or 2 polarization). The result is an altered state of functional immune maturation and altered responses also to unrelated pathogen exposure.^4^

The involvement of similar phenomena and mechanisms to explain the tolerogenic response of infants and children toward SARS-CoV-2 infection has been invoked. In this study, we analyze a cohort of newborns born to mothers with SARS-CoV-2 infection, for which we determine the infection status, the presence of SARS-CoV-2-specific IgM, IgG and IgA and the count and proportion of lymphocyte and natural killers (NK) subpopulations, and hypothesize a possible influence of the maternal infection status on the newborn immune system.

## METHODS

### Patient Population

We conducted a retrospective analysis of clinical records and laboratory data of neonates from SARS-CoV-2-infected mothers, born in, or transferred to, Luigi Sacco Hospital (Milan, Italy) from March 16 to November 1, 2020. Only term and late preterm newborns were included. Management of newborns was in accordance with hospital protocols and guidelines provided by the Italian Neonatal Society.

The maternal and neonatal infection states for SARS-CoV-2 were laboratory determined by means of real-time reverse transcription polymerase chain reaction (RT-PCR) tests performed on a nasopharyngeal swabs (NPS). The women enrolled had an active SARS-CoV-2 infection at the time of delivery and all were tested 24 hours before entering the delivery room or upon entering. All the neonates were tested within 24 hours from the birth and before the hospital discharge, usually occurred at 3rd day of life. Immunophenotypes of lymphocyte and NK subsets and SARS-CoV-2-specific IgA, IgM and IgG antibodies were evaluated on blood samples collected within the first 24 hours from birth. Our cohort of newborns was divided into groups according to seropositivity or not for IgG(IgG+/−) and positivity or not of the NPS (NPS+/−).

The study was approved by the ethical committees of the coordinating center in Milan (protocol number 2020/ST/061).

### Flow Cytometry Immunophenotyping

Lymphocytes subsets were measured by AQUIOS CL flow cytometer (Beckman Coulter, Miami, FL) using fresh blood sample.^5^ A customized Ab mix was made for this clinical purpose. This panel consisted of CD3, CD4, CD8, CD19 and CD3−/CD16+/CD56+ (Beckman Coulter). Absolute leukocyte count was based on an electronic volume measurement.

### SARS-CoV-2-specific Antibodies

Serum was tested for anti-SARS-CoV-2-specific IgG, IgA and IgM using the semiquantitative anti-SARS-CoV-2 ELISA (Euroimmun, Lübeck, Germany) test, according to manufacturer’s instructions. Specifically, 10 µL of each serum was diluted 1:10 in sample buffer, and 100 µL of diluted serum was incubated into individual microplate wells coated with a recombinant S1 domain of the SARS-CoV-2 spike protein for IgG and IgA and a modified nucleocapsid protein that only contains diagnostically relevant epitopes for IgM. Results were acquired semiquantitatively by a ratio (optical density sample/optical density calibrator) and interpreted as follows: <0.8, negative; ≥0.8 to <1.1, borderline; ≥1.1, positive.

### Statistical Analysis

The Student *t* test, the χ² method and Fisher exact test were done when appropriate for statistical analysis to compare continuous and categorical variables. A *P* value <0.05 was chosen as cutoff for significance. Data were analyzed with Excel and graphs were created with Prism 9 (GraphPad Software, San Diego, CA).

## RESULTS

We enrolled 75 newborns from mothers with SARS-CoV-2 infection present during childbirth. Ten (13.3%) of 75 neonates tested positive at NPS for SARS-CoV-2 at birth. All of them were asymptomatic. All the newborns were SARS-CoV-2-IgA and IgM seronegative. Eighteen of 59 (30%) were SARS-CoV-2-IgG seropositive, only one of which with NPS for SARS-CoV-2.

**FIGURE 1.**
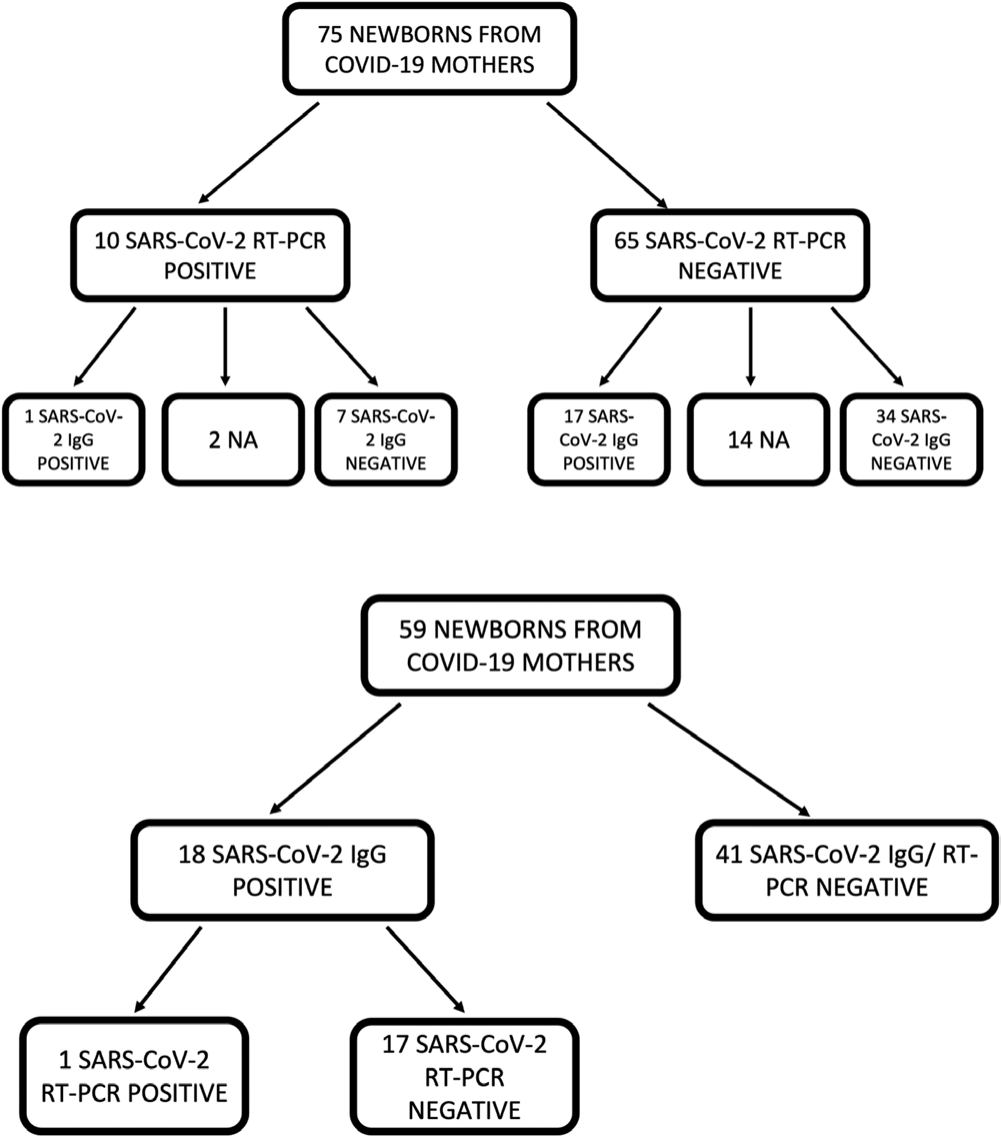
Flow chart of the study cohort.

We thus divided our cohort into 4 groups: newborns tested positive (NPS+, 10 of 75) and negative (NPS−, 65 of 75) upon RT-PCR examination and newborns IgG seropositive (IgG+, 18 of 59) and seronegative (IgG−, 41 of 59) (Fig. 1).

In the whole cohort and in the defined groups, we determined (Table 1): (1) the white blood cells (WBC) count; (2) the absolute count and relative size of T cells (CD3+), CD4+ T cells, CD8+ T cells, CD4/CD8 ratio, B cells (CD33+) and NK (CD3−, CD16+, CD56+).

**TABLE 1.** Immunophenotyping in Newborn From SARS-CoV-2-infected Mothers

Test Items	All Newborns				SARS-CoV-2-IgG Seronegative Newborns (n = 41)				SARS-CoV-2-IgG seropositive newborns (n = 17)				IgG-positive vs. IgG-negative newborns		RT-PCR NPS-negative newborns (n = 65)				RT-PCR NPS-positive newborns (n = 10)				NPS-positive vs. NPS-negative newborns	
	Mean		(95% CI)		Mean		(95% CI)		Mean		(95% CI)		*P* Value		Mean		(95% CI)		Mean		(95% CI)		*P* Value	
	# (10^9^/L)	%	#	%	# (10^9^/L)	%	#	%	# (10^9^/L)	%	#	%	%	#	# (10^9^/L)	%	#	%	# (10^9^/L)	%	#	%	%	#
WBC count	17.41	-	16.06–18.75	-	17.76	-	16–19.59	-	16.24	-	13.25–18.23	-	-	0.39	17.82	-	16.35–19.30		14.7	-	11.91–17.49	-	-	0.07
CD3 T cells	2.95	75.67	2.72–3.18	74.19–77.25	2.93	74.41	2.67–3.18	73.13–75.70	3.16	79.56	2.41–3.91	76.55–82.58	**0.005**	0.56	2.96	75.9	2.70–3.22	74.25–77.54	2.88	74.21	2.37–3.39	71.18–77.24	0.35	0.78
CD4 T cells	2.18	55.92	2.01–2.36	54.21–57.62	2.17	55.13	1.98–2.36	53.12–57.13	2.31	57.99	1.75–2.87	55.21–60.78	0.11	0.64	2.21	56.47	2.01–2.40	54.60–58.35	2.04	52.35	1.70–2.38	49.14–55.56	**0.04**	0.42
CD8 T cells	0.74	18.58	0.66–0.82	17.47–19.68	0.73	18.34	0.64–0.83	16.69–19.98	0.81	20.17	0.61–1.02	18.63–21.71	0.11	0.5	0.73	18.3	0.64–0.81	17.16–19.44	0.81	20.34	0.61–1.01	16.66–24.02	0.32	0.46
CD4/CD8	3.24	-	2.98–3.51	-	3.34	-	2.94–3.74	-	2.96	-	2.67–3.25	-	0.19	-	3.31	-	3.02–3.60	-	2.83	-	2.16–3.50	-	0.22	
CD19 B cells	0.61	12.97	0.45–0.77	11.94–14.01	0.72	13.48	0.45–1.00	12.13–14.83	0.44	11.55	0.33–0.56	9.85–13.25	0.09	0.06	0.63	13.03	0.45–0.81	11.90–14.16	0.5	12.62	0.37–0.63	10.00–15.24	0.78	0.25
NK (CD3−,CD16+,CD56+)	0.3	7.93	0.26–0.34	6.95–8.90	0.32	8.23	0.26–0.38	7.10–9.35	0.23	6.71	0.18–0.29	4.91–8.50	0.17	**0.04**	0.3	7.82	0.25–0.34	6.80–8.85	0.34	8.79	0.18–0.50	5.35–12.22	0.61	0.61

Data are expressed in mean (10^9^/L) and percentage where appropriate. Fisher exact test was applied, a *P* value <0.05 was considered significant (in bold).

Considering the whole cohort, the mean WBC count was 17.41 × 10^9^/L [95% confidence interval (CI): 16.06–18.75]; mean of CD3 T cells was 2.95 × 10^9^/L (95% CI: 2.72–3.18) accounting for 75.67% (95% CI: 74.19–77.25) of total WBC; mean of CD4 T cells was 2.18 × 10^9^/L (95% CI: 2.01–2.36), accounting for 55.92% (95% CI: 54.21–57.62) of total WBC; mean of CD8 T cells was 0.74 × 10^9^/L (95% CI: 0.66–0.82), accounting for 18.58% (95% CI: 17.47–19.68) of total WBC; mean of CD4/CD8 T cells ratio was 3.24 (95% CI: 2.98–3.51); mean of B cells was 0.61 × 10^9^/L (95% CI: 0.45–0.77), accounting for 12.97% (95% CI: 11.94–14.01) of total WBC; mean of NK cells was 0.3 × 10^9^/L (95% CI: 0.26–0.34), accounting for 7.93% (95% CI: 6.95–8.90) of total WBC. The count and proportion of the analyzed immune subsets were all in the normal range according to age, compared with data already present in literature.^6^

**FIGURE 2.**
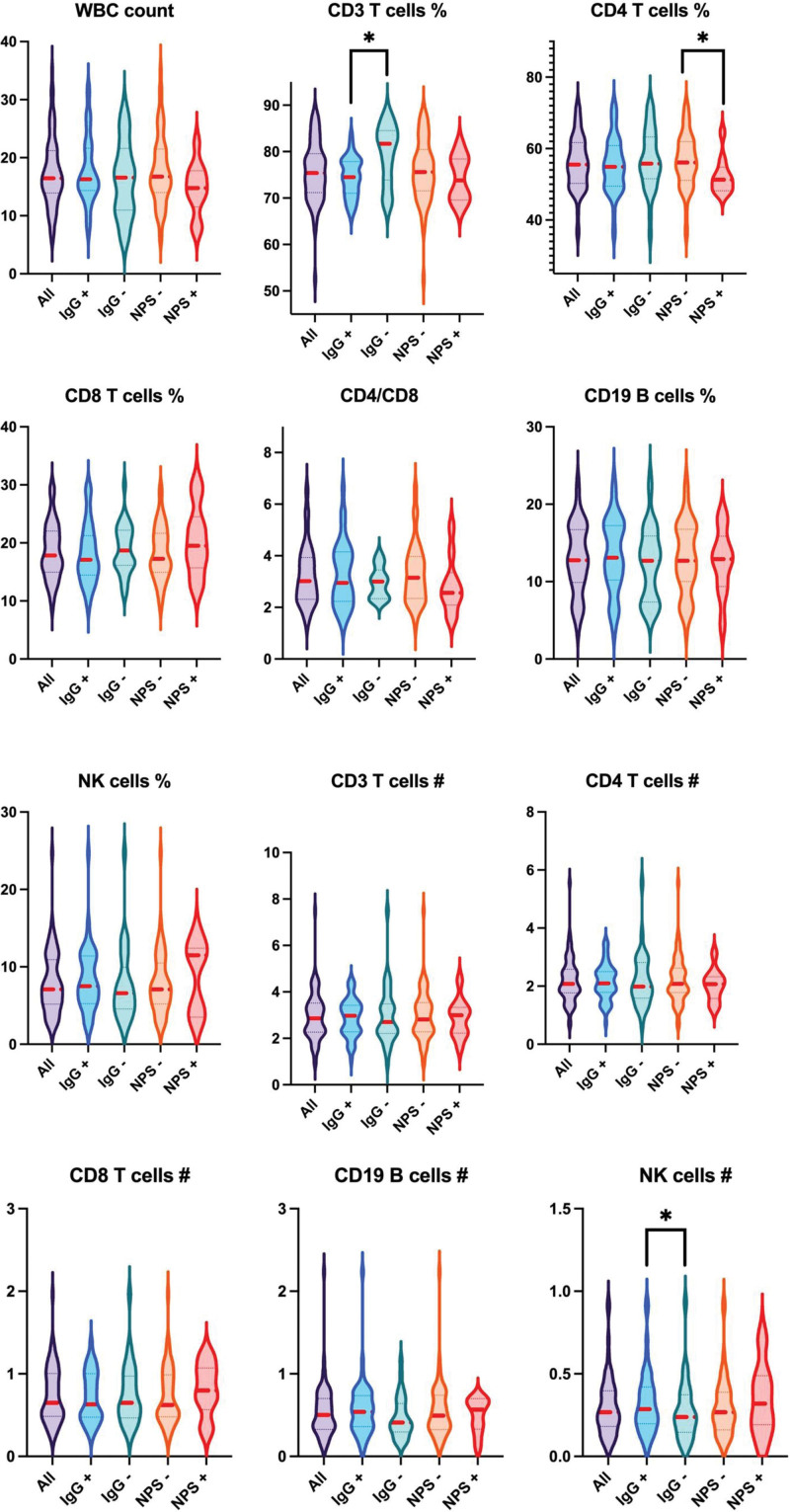
Violin plots of lymphocyte subpopulations. Data are expressed in percentage and 10^9^/L as mean. *P value >0.05.

When comparing IgG+ and IgG− newborns for all the previous parameters (Fig. 2), CD3 T cells resulted relatively higher in the IgG+ group (*P* = 0.005); conversely, NK cells in absolute size were higher in IgG− group (*P* = 0.04); no other differences were found for remaining subpopulations. Comparing, finally, NPS+ with NPS− groups, a higher CD4 T cells percentage characterized the NPS− group (*P* = 0.04); no other differences were found between these groups.

## DISCUSSION

It is already recognized that the functional maturation of neonatal immunity can be modulated by external factors, as live vaccines and maternal infections.^4^ For example, it is known that in utero exposure to maternal malarial infection results in an expansion of effector and regulatory T cells in the fetus; moreover, the timing of exposure plays a key role in determining the polarization of the fetal immune response.^7^ Infection with *Plasmodium falciparum* at delivery has been shown to determine changes in the immune system of the newborn that can still be evidenced 12 months of life and consist in a relative increase of CD8 lymphocytes, with parallel decrease of CD4 cells in the postnatal period.^8^

Maternal HIV infection also leads to an alteration of the fetal immune system, even in the case of nonvertical transmission of the infection to the newborn.^9^

It is still to be determined which implications derive from the maternal SARS-CoV-2 infection on the fetal immune system, and subsequently, in the infant.

In a recent study, changes in gene expression in polymorphonuclear cells of newborns born to mothers infected with SARS-CoV-2 were addressed. The authors reported the upregulation of the interferon-stimulating genes, and the activation and depletion of NK cells. When comparing their cohort of enrolled infants, none of whom acquired SARS-CoV-2 infection, to healthy controls, they noted an expansion of the T cell subset.^10^

To the best of our knowledge, our study is among the first ones to compare immunophenotyping in newborns from SARS-CoV-2-infected mothers, according to the neonatal IgG anti-Spike seroconversion and infection status.

Presence of specific antibody against SARS-CoV-2 in our newborn cohort is not informative about any congenital or postnatal infection as we know IgG could have been transferred from mother to child via placenta.

We found a significant expansion of the T cell (CD3+) subset in the IgG+ group; this could be interpreted as the result of immune effects triggered by trained immunity in these newborns. It should be emphasized that no specific IgM and IgA were found in any newborn, in accordance with the hypothesis (to be confirmed, anyway, by the maternal placentas) of noncongenital infection.

Conversely, the decrease detected in CD4+ T cells for NPS+ neonates agrees with published data, suggesting a possible immunopathogenic pathway of SARS-CoV-2 infection,^11^ even if it is difficult to conclude that the decrease in CD4 can certainly be caused by an infection.

Our data can be supplemented by those present in a recent study carried out on a cohort of newborns born to a mother with SARS-CoV-2 infection^12^; in particular, the authors did not find any significant differences in the analysis of lymphocyte subpopulations related to the timing of maternal disease onset. Similarly, in our cohort, they did not report any difference in the count and proportion of immune cell distribution, except for NK cells.

Our study has several limitations: no data on cytokine and immune gene expression were collected; we were not able to enroll a control group as we were a SARS-CoV-2 hub (only COVID-19 patients were allowed to our center); we unfortunately missed the clinical data of the mothers, this could give us the chance to correlate them to newborns data; this is a retrospective analysis and as such some data were not collected with the intention of being subsequently analyzed and published.

Our study is one of the few that focuses on the immune system of the newborn from a SARS-CoV-2-infected mother; with our data, we bring to light how the maternal infection, even if acquired in the terminal period of pregnancy, does not cause a congenital infection and seems to stimulate the fetal immune system directing it toward a presumably protective expansion of the T subset as seen also in infection from other in utero exposures.

In our cohort, there is no evidence of unbalanced differentiation of lymphocyte subsets; maternal infection by SARS-CoV-2 in the peripartum period does not apparently cause dysfunction in newborn immunophenotype, resulting in no increased risk of congenital or perinatal infection. This article has the purpose to point out that like other infections already studied in the past, even if SARS-CoV-2 infection is acquired so late in pregnancy, it can generate effects on the baby: these can be positive, such as short-term protection by the humoral immune response from the infection itself or the influence on the newborn’s immune system to react against different pathogens in accordance with the theory of trained immunity.
